# Supplementary material for: Compressive stress triggers fibroblasts spreading over cancer cells to generate carcinoma in situ organization
Source: Commun Biol. 2024 Feb 15;7:184. doi: 10.1038/s42003-024-05883-6 (PMC10869726; doi:10.1038/s42003-024-05883-6)
Supplement: Supplementary file 2 — Supplementary information [file 42003_2024_5883_MOESM2_ESM.pdf]

Supplementary Information

Supplementary figures

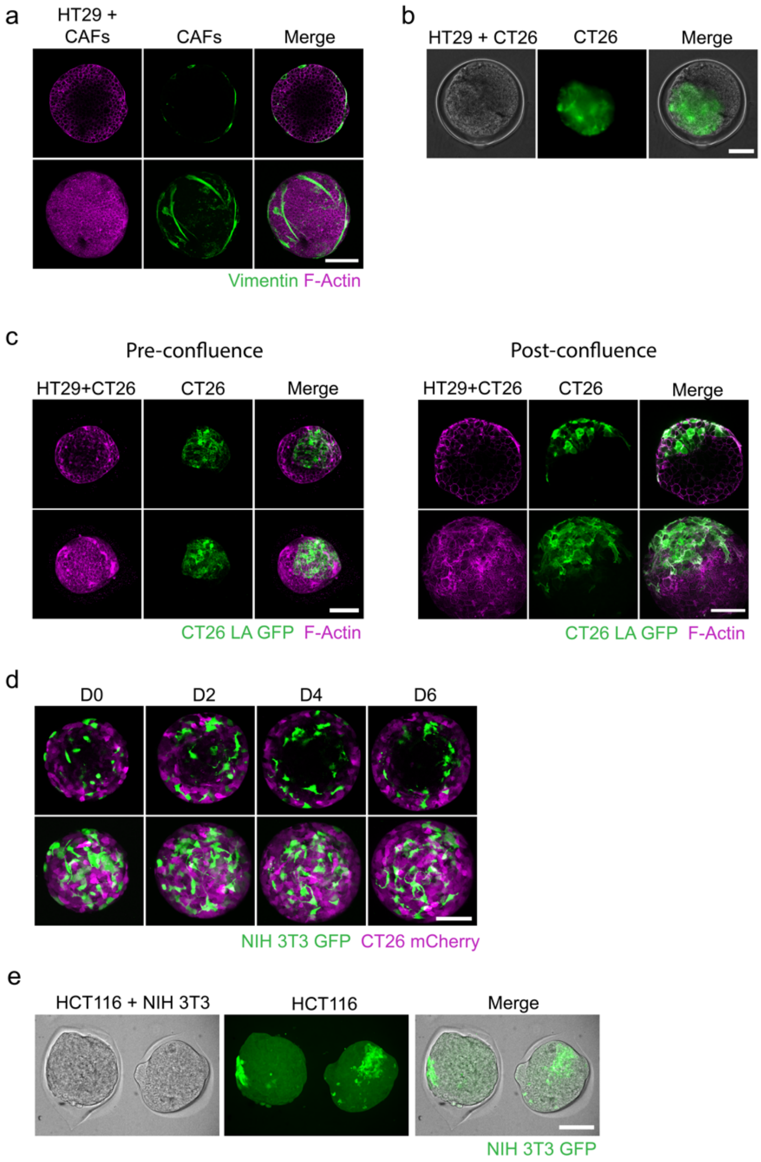

Suppl. Figure 1. Co-culture of epithelial and mesenchymal cells

a. Confocal images of co-culture of primary non-immortalized human CAFs (vimentin, green) and cancer cells (HT29) after confluency. All cells labeled for F-actin (phalloidin, magenta). Scale bar, 150 µm.

b. Phase contrast image of co-culture of HT29 (unlabeled) and CT26 cells (green, LifeAct-GFP) pre-confluence. Scale bar, 100 µm.

c. Co-culture of HT29 (unlabeled) and CT26 cells (green, LifeAct-GFP). F-actin (phalloidin, magenta). Left panel, pre-confluence. Scale bar, 80 µm. Right panel, pre-confluence. Scale bar, 100 µm.

d. Co-culture of NIH3T3 expressing GFP (green) and CT26 cells expressing mCherry (magenta) over 6 days. Scale bar, 200 µm.

e. Co-culture of NIH3T3 expressing GFP (green) and HCT116 cells (unlabeled). Phase contrast images overlayed with fluorescent images of NIH3T3. Scale bar, 200 µm.

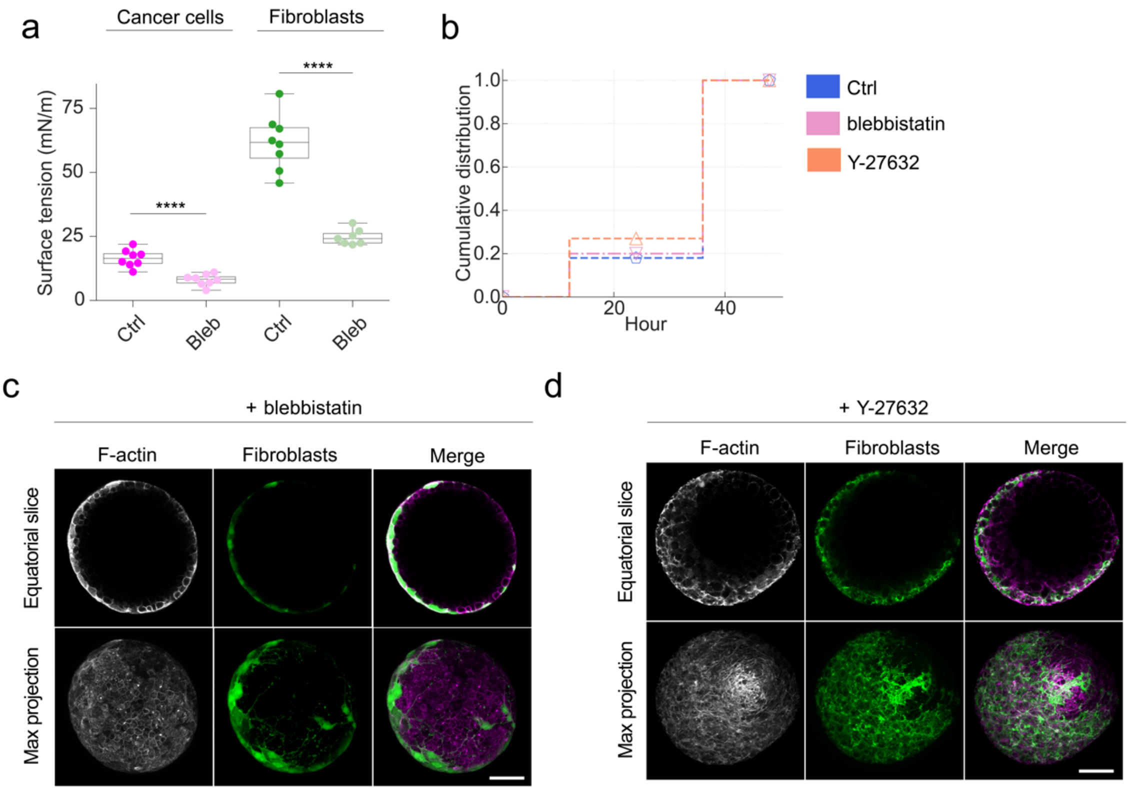

Supp. Fig. 2. Inhibition of myosin with blebbistatin reduce surface tension

a. Individual (dots) surface tension measurements on cancer cells (red) and fibroblasts (green) spheroids treated with blebbistatin or DMSO (Control). One-way ANOVA test. n = 31 capsules.

b. Percentage of capsules in which fibroblasts envelop spheroids of cancer cells over time. Capsules were either untreated Control (blue) or treated with blebbistatin (brown) and Y27632 (orange). Drugs were applied about 15h before the confluence. T=0 corresponds to the confluent stage. n=60 capsules.

c-d. Confocal images of co-culture after fibroblast spreading, treated with blebbistatin (c) or Y27632 (d) (day 12). Fibroblasts expressed GFP (green), cancer cells are unstained, F-actin (phalloidin, magenta). Scale bars: 20 µm.

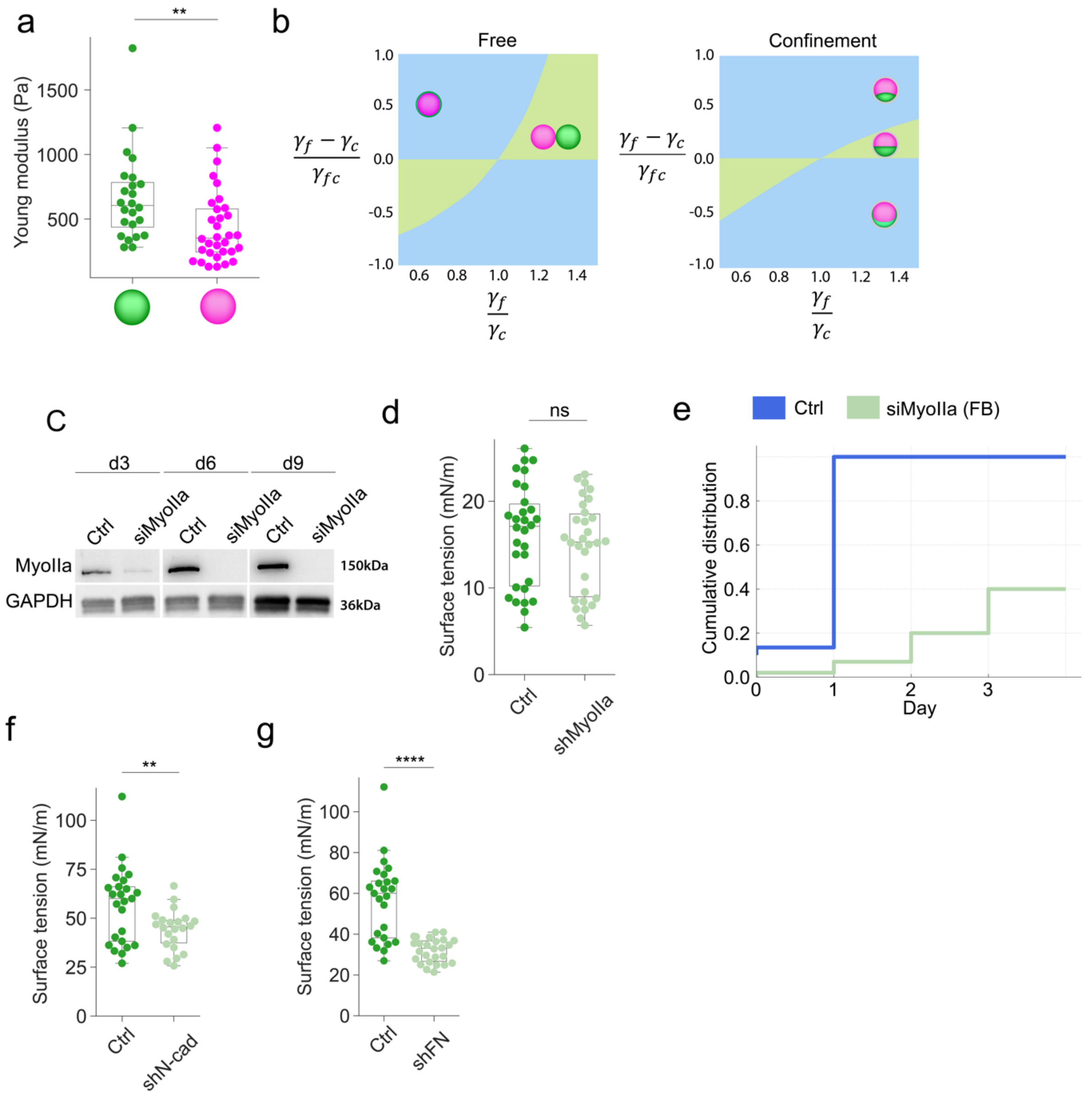

Suppl. Fig. 3. Differential surface tension cannot explain fibroblast spreading

a. Young's modulus of (green) fibroblasts and cancer cells (magenta) spheroids measured using the microplate technique. n=24 spheroids for fibroblasts and n=32 spheroids for cancer cells from N=3 independent experiments.

b. The surface-energy model predictions on the relative likelihood of different morphologies in the space of the relative surface tensions of the fibroblasts and cancer cells, and the ratio of the difference between these surface tensions and the interfacial tension. Left, the region of phase space where the core-shell morphology is preferred vs. where the side-by-side morphology in the absence of confinement (eqn. S4). Right, the corresponding regions for the Janus and side-by-side morphologies in confinement.

c. Western blot showing myosinIIA expression level in control fibroblasts (Ctrl, transfected with scrambled siRNA) and myosinII-depleted fibroblasts (shMyosinIIA), 3, 6 and 9 days after transfection. GAPDH is used as a loading control.

d. Individual (dots) surface tension measurements on control and myosinIIA-depleted fibroblast spheroids growing without confinement. One-way ANOVA test. n = 59 capsules, from N=3 independent experiments

e. Percentage of capsules in which control or myosinIIA-depleted fibroblasts envelop spheroids of cancer cells over time. T=0 corresponds to the confluent stage. n≥40 capsules per condition.

f. Individual (dots) surface tension measurements on control and N-cadherin-depleted fibroblast spheroids growing without confinement. One-way ANOVA test. n = 47 capsules, from N=3 independent experiments

g. Individual (dots) surface tension measurements on control and fibronectin-depleted fibroblast spheroids growing without confinement. One-way ANOVA test. n = 51 capsules, from N=3 independent experiments

Western Blot - shNcadherin NIH3T3

a

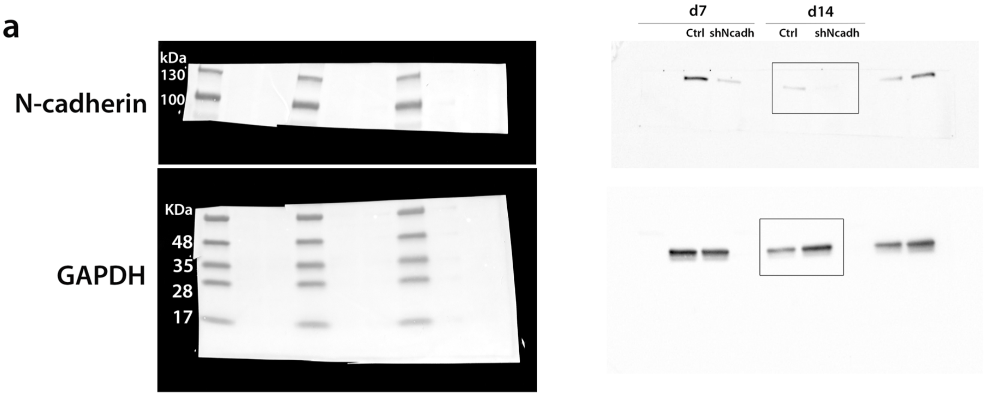

Western Blot - shNMHIIa HT29

b

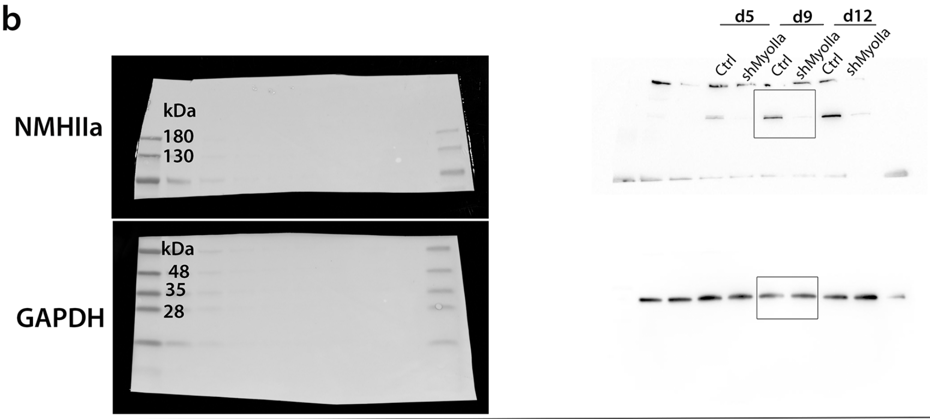

Western Blot - siNMHIIa NIH3T3

c

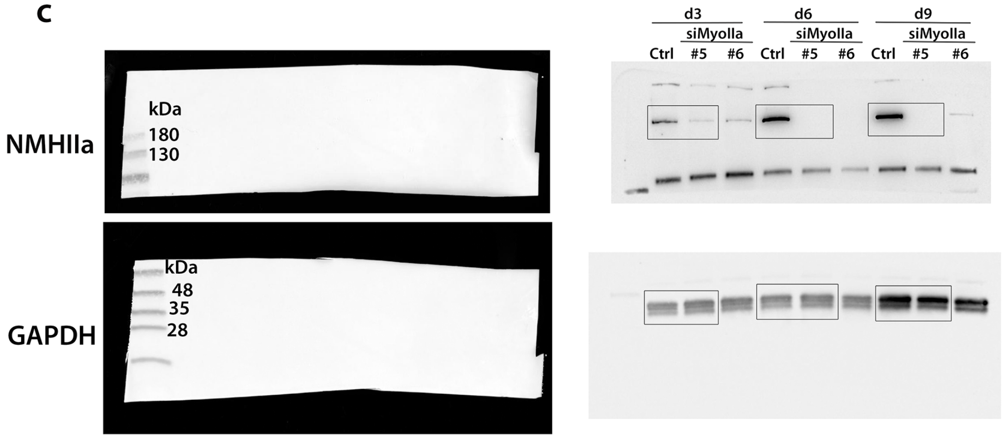

Western Blot - siFN NIH3T3

d

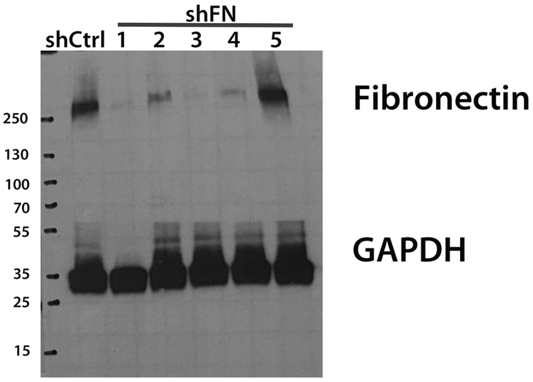

Suppl. Fig. 4. Uncropped western blots

Uncropped western blots show molecular weight markers on the left and specific bands on the right. Boxed regions show part of the blot presented in the main figure. a. Blot from figure 3a; b. Blot from figure 4f; c. Blot from suppl. figure 3c. ; d. Blot from figure 3c.

Supplementary Notes

Supplementary Note 1. Simplified surface energy model for side-by-side and core shell morphologies

We first consider the following side-by-side and core shell morphologies shown in Suppl. Fig. 2b. We model cellular aggregates as two spheroids with minimal overlap for the side-by-side configuration, and a spheroid with a concentric core for the core-shell configuration. No confining constraints are assumed. The total surface energy of the two morphologies [19] are:

$$E_1 = 4\pi * (r_c^2 \gamma_{fc} + R^2 \gamma_f) \quad \text{(S1)}$$

$$E_2 = 4\pi * \left(r_c^2 \gamma_c + r_f^2 \gamma_f\right), \quad \text{(S2)}$$

where r_f is the radius of the fibroblast aggregate, r_c is the radius of the cancer cell aggregate, and R is the radius of the spheroid, γ_{fc} is the interfacial tension between the two types of cells, γ_c is the surface tension of the cancel cell aggregate (in culture medium) and γ_f is the surface tension of the fibroblast aggregate. Using the above equations (S1) & (S2), we obtain the surface energy difference of the spheroids between Morphology 1 & 2 to be:

$$\frac{E_1 - E_2}{4\pi\gamma_c {r_f}^2} = \left(\frac{N_c}{N_f}\right)^{\frac{2}{3}} \frac{\gamma_{fc} - \gamma_c}{\gamma_c} + \left(\left(\frac{N_f + N_c}{N_f}\right)^{\frac{2}{3}} - 1\right) \frac{\gamma_f}{\gamma_c} \quad \text{(S3)}$$

where N_c and N_f are the respective numbers of cancer cells and fibroblasts in the cellular aggregates. Here, we assume all the cells have the same size, thus $r_c/r_f = (N_c/N_f)^{1/3}$ and $\frac{R}{r_f} = \left(\frac{N_c+N_f}{N_f}\right)^{\frac{1}{3}}$.

Further simplifying equation (S3), we have:

$$\frac{E_1 - E_2}{4\pi\gamma_c {r_f}^2} = \left[\left(\frac{1}{k}\right)^{\frac{2}{3}} \left(\frac{\alpha - 1}{\omega} - 1\right)\right] + \left[\left(\left(1 + \frac{1}{k}\right)^{\frac{2}{3}} - 1\right) \alpha\right] \quad \text{(S4)}$$

where $k = \frac{N_f}{N_c}$, $\alpha = \frac{\gamma_f}{\gamma_c}$, and $\omega = (\gamma_f - \gamma_c)/\gamma_{fc}$. In generating Suppl. Fig. 2b (left) in the main manuscript, we have assumed k=1, i.e. there are the same number of fibroblasts and cancer cells in the aggregates.

Of note, this model presumes the spheroids behave as liquids, in line with most prevalent models, and thus does not allow to introduce per se elastic parameters such as Young's moduli that characterize solids. The liquid-like behavior is supported here by the observation of the dewetting process after capsule dissolution, for instance. A theoretical approach treating the

spheroids as viscoelastic liquids could be more appropriate, but we are unaware of any such analytical framework that could be simply extended to assess a potential difference between free and confined situations. In the following section, we conserve the initial liquid-like hypothesis and propose a more detailed calculation.

Supplementary Note 2. Energy Model of Confined Janus Morphology

Geometry of Confined Janus: In this section, we develop the expressions for the geometry and energy of a two-component system (denoted by subscripts f and c) of immiscible fluids confined to a spherical volume with radius R_0. Like the previous analysis, we assume that the long timescale behavior of the two-cell type system is well approximated by a fluid, such that only surface tension (rather than elastic forces) dominates the behavior. We will show that the system is completely parameterizable by the contact angle θ(which we measure from within the cancer cell phase, see Suppl. Fig. 5) and the volume fraction of fibroblasts

$\phi = \frac{V_f}{(V_f+V_c)} = \frac{k}{k+1}$, (S5)

(where k is the volume ratio defined in SI). However, it is initially convenient to introduce the radius of curvature of the interface between the two phase R and the opening angle β, which defines the location of the contact line, see Suppl. Fig. 5 and [48].

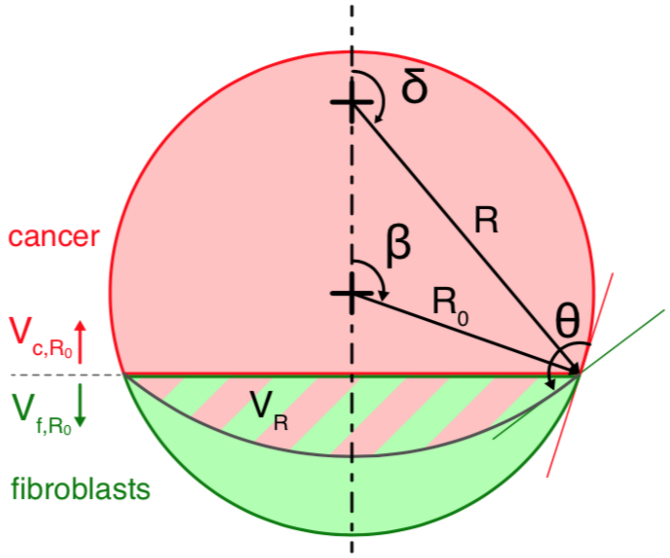

Suppl. Fig. 5: Schematic of a confined Janus morphology with key geometric variables and parameters labeled.

Our first goal is to write expressions for the volume of each phase. Each can be broken into two spherical caps: one with a radius R_0 given by the confining capsule and the other with a radius of the interface between the two phases R. The second volume accounts for the protrusion of one phase into the other and thus adds to one volume at the expense of the other. The expressions for the volume of each phase are therefore

$V_f = V_{f,R_0} + V_R$, (S6)

$V_c = V_{c,R_0} - V_R$, (S7)
where the first components for each are
$V_{f,R_0} = \frac{4}{3}\pi R_0^3 - \frac{1}{3}\pi R_0^3(2 - 3cos\beta + cos^3\beta)$, (S8)
$V_{c,R_0} = \frac{1}{3}\pi R_0^3(2 - 3cos\beta + cos^3\beta)$, (S9)
and the last contribution
$V_R = \frac{1}{3}\pi R^3(2 - 3cos\delta + cos^3\delta)$, (S10)
is the protruding portion (hatched green-red region in Suppl. Fig. 5). We've introduced a new opening angle δ for the spherical section of the interface, which can be related to the opening angle and contact angle through $\delta = \theta - \beta$. Finally, the radius of curvature of the interface can also be written in terms of these variables $R = sin\beta/sin(\theta - \beta)$. We note that this expression naturally changes the sign of R (whether the interface is convex or concave) depending on the contact angle and the contact line location. Consequently, the sign of V_R also changes. Taken together, we can plot ϕ as a function of (β, θ), Suppl. Fig. 6. Importantly, we note that for any given contact angle θ, all volume fractions ϕ are accessible; this means that the geometry of the two-phase-system is always well-defined.

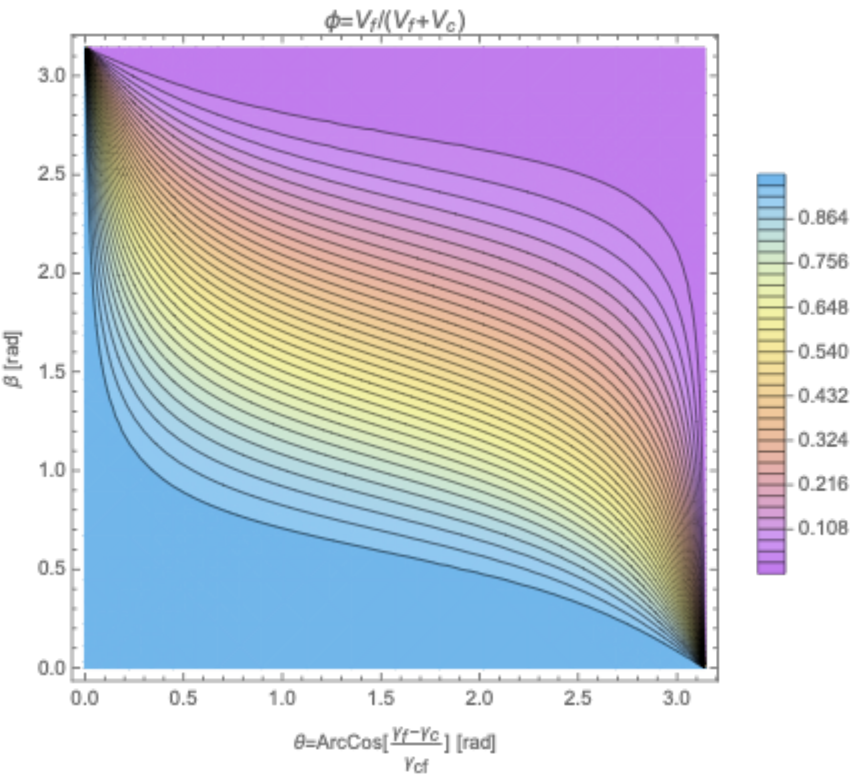

Suppl. Fig. 6: Volume fraction of fibroblasts (contours) as a function of contact line position β and contact angle θ.

Energy of configurations: In this section we compare the energy of the confined Janus configuration to the other limiting morphologies (enwrapped and side-by-side). The total surface energy for the Janus configuration is given by
$E_{Janus} = \pi R_0^2 \left[\gamma_{cf} sec^2(\beta) sec^2\left(\frac{\beta-\theta}{2}\right) + 2\left(\gamma_f + \gamma_c + (\gamma_f - \gamma_c)cos(\beta)\right)\right]$, (S11)

where the opening angle β depends on the contact angle and volume fraction and must be found numerically by solving the transcendental equation for volume ration, Eqn. S6. We compare Eqn. S11 and Eqn. S1 (enwrapped state) for various volume fractions in Suppl. Fig. 7.

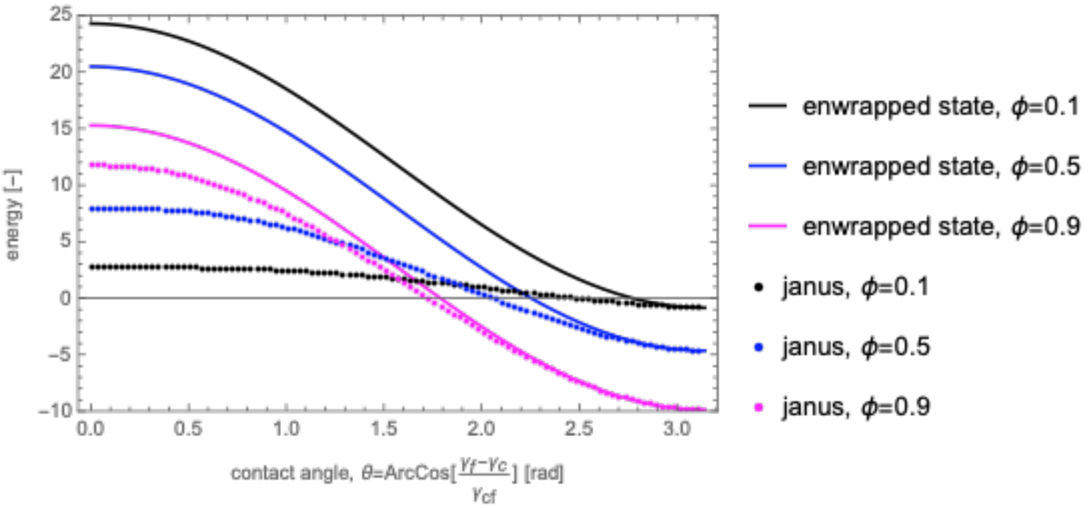

Suppl. Fig. 7: We plot the energy of both the fully engulfed state and the Janus morphology as a function of contact angle.

Without any loss in generality, we define the surface energies in terms of the contact angle such that $\gamma_{cf} = 1, \gamma_c = 0$ *and* $\gamma_f = cos\theta$. *The figure shows that, so long as a finite contact angle* $\theta \in [0, \pi]$, *can be defined, the Janus configuration is the energetic minima. When the outer, fibroblast fully wets the capsule walls* ($\theta = \pi$), *the two states are geometrically identical (for all volume ratios).*

In the main text, we compare eqn. S11 to the side-by-side configuration eqn. S3; subtracting these energies and non-dimensionalizing gives

$$\frac{E_{Janus}-E_2}{4\pi R_0^2\gamma_c} = \frac{1}{2}\left(1 - \frac{2}{(1+k)^{\frac{2}{3}}} + \alpha - 2\alpha\left(\frac{k}{k+1}\right)^{\frac{2}{3}} + (\alpha - 1)cos(\beta)\right) + \frac{(\alpha-1)}{4}sec(\theta)sec^2(\beta)sec^2\left(\frac{\beta-\theta}{2}\right), \text{(S12)}$$

where we've substituted the volume fraction ϕ for the volume ratio *k*.

Supplementary Note 3. Discussion of Measured Surface Tension Values

We note that in the experiments, the difference in surface energies between fibroblasts and cancer cells decreases but did not change signs upon confinement and subsequent confluence. In terms of contact angle, this is identical to the contact angle tending towards 90 [deg] but not surpassing it. This contradicts the observation that the fibroblasts "wet" the capsule (by the sign convention in Suppl. Fig. 5, perfect wetting of the fibroblasts occurs at 180 [deg]). We speculate as to the cause of the mismatch between observed morphology and measured surface tension. (1) Firstly, the measured surface energies correspond to the cell-aqueous interface, not the cell-capsule interface. It is possible that these energies become different when fibronectin is expelled from the fibroblasts. We note that if both cancer cells and fibroblast energies are equally offset

by the presence of the capsule, then these contributions will cancel out. So, for this picture to hold, the cell types must be differentially impacted by the change in material. (2) Secondly, the timescale over which the physiological changes that alter the surface tension relaxes may be rapid enough to evade measurement. This assumes that the local change in energies is rapid compared to the overall rearrangement. (3) Lastly, the thinness of the fibroblast layer may mean that the measurements were influenced by the deformation of the cancer-fibroblast interface. This would lead to an overestimate of the fibroblast energy.
