# Supplementary material for: Compressive stress triggers fibroblasts spreading over cancer cells to generate carcinoma in situ organization
Source: Commun Biol. 2024 Feb 15;7:184. doi: 10.1038/s42003-024-05883-6 (PMC10869726; doi:10.1038/s42003-024-05883-6)
Supplement: Supplementary file 3 — Description of Additional Supplementary Files [file 42003_2024_5883_MOESM3_ESM.pdf]

Description of Additional Supplementary Files

File name: Supplementary Movie 1
Description: Time-lapse imaging of co-culture of HT29 cancer cells and GFP expressing NIH 3T3 fibroblasts (green) in alginate capsules. Time t = 0 corresponds to encapsulation of cells. Merge of phase contrast and epifluorescent images. Time in hours:min. Scale bar, 100 µm.

File name: Supplementary Movie 2
Description: Time-lapse imaging of co-culture of HT29 cancer cells and GFP expressing NIH 3T3 fibroblasts (green) after removal of alginate capsules. Time t = 0 corresponds to capsule dissolution, thus release of the confinement. Time in hours:min. Scale bar, 100 µm.

File name: Supplementary Movie 3
Description: Two-photon live imaging of a co-culture of cancer cells stained by membrane die FM4-64 (red) and fibroblasts expressing GFP (green) at the onset of confluency. One optical slice at the equatorial plane. Scale bar: 50µm.

File name: Supplementary Movie 4
Description: Two-photon live imaging of a co-culture of cancer cells stained by membrane die FM4-64 (red) and fibroblasts expressing GFP (green) at the onset of confluency. Maximal projection. Scale bar: 50µm.

File name: Supplementary Data 1
Description: Source data behind the graphs in the manuscript.
