# Supplementary material for: Compressive stress triggers fibroblasts spreading over cancer cells to generate carcinoma in situ organization
Source: Commun Biol. 2024 Feb 15;7:184. doi: 10.1038/s42003-024-05883-6 (PMC10869726; doi:10.1038/s42003-024-05883-6)
Supplement: Supplementary file 9 — Reporting Summary [file 42003_2024_5883_MOESM9_ESM.pdf]

nature portfolio

Corresponding author(s): Danijela Vignjevic & Pierre Nassoy

Last updated by author(s): Dec 10, 2023

Reporting Summary

Nature Portfolio wishes to improve the reproducibility of the work that we publish. This form provides structure for consistency and transparency in reporting. For further information on Nature Portfolio policies, see our Editorial Policies and the Editorial Policy Checklist.

Statistics

For all statistical analyses, confirm that the following items are present in the figure legend, table legend, main text, or Methods section.

n/a	Confirmed	
☐	☒	The exact sample size (*n*) for each experimental group/condition, given as a discrete number and unit of measurement
☐	☒	A statement on whether measurements were taken from distinct samples or whether the same sample was measured repeatedly
☐	☒	The statistical test(s) used AND whether they are one- or two-sided<br>*Only common tests should be described solely by name; describe more complex techniques in the Methods section.*
☒	☐	A description of all covariates tested
☐	☒	A description of any assumptions or corrections, such as tests of normality and adjustment for multiple comparisons
☐	☒	A full description of the statistical parameters including central tendency (e.g. means) or other basic estimates (e.g. regression coefficient) AND variation (e.g. standard deviation) or associated estimates of uncertainty (e.g. confidence intervals)
☐	☒	For null hypothesis testing, the test statistic (e.g. *F*, *t*, *r*) with confidence intervals, effect sizes, degrees of freedom and *P* value noted<br>*Give P values as exact values whenever suitable.*
☒	☐	For Bayesian analysis, information on the choice of priors and Markov chain Monte Carlo settings
☒	☐	For hierarchical and complex designs, identification of the appropriate level for tests and full reporting of outcomes
☒	☐	Estimates of effect sizes (e.g. Cohen's *d*, Pearson's *r*), indicating how they were calculated

Our web collection on statistics for biologists contains articles on many of the points above.

Software and code

Policy information about availability of computer code

Data collection	Image acquisition was performed using Metamorph software for videomicroscopy and spinning disk confocal microscopy and Leica SP8 two photon confocal microscope controlled through the commercial LAS software.
Data analysis	Software for data analysis are cited in the methods section and include: ImageJ/FIJI for image analysis and Python.

For manuscripts utilizing custom algorithms or software that are central to the research but not yet described in published literature, software must be made available to editors and reviewers. We strongly encourage code deposition in a community repository (e.g. GitHub). See the Nature Portfolio guidelines for submitting code & software for further information.

Data

Policy information about availability of data

All manuscripts must include a data availability statement. This statement should provide the following information, where applicable:

- Accession codes, unique identifiers, or web links for publicly available datasets
- A description of any restrictions on data availability
- For clinical datasets or third party data, please ensure that the statement adheres to our policy

All data in the manuscript will be made available upon reasonable request.

Human research participants

Policy information about studies involving human research participants and Sex and Gender in Research.

Reporting on sex and gender	NA
Population characteristics	NA
Recruitment	NA
Ethics oversight	NA

Note that full information on the approval of the study protocol must also be provided in the manuscript.

Field-specific reporting

Please select the one below that is the best fit for your research. If you are not sure, read the appropriate sections before making your selection.

☒ Life sciences ☐ Behavioural & social sciences ☐ Ecological, evolutionary & environmental sciences

For a reference copy of the document with all sections, see nature.com/documents/nr-reporting-summary-flat.pdf

Life sciences study design

All studies must disclose on these points even when the disclosure is negative.

Sample size	Sample size was not predetermined. The number of samples used in each experiment was determined based on variation in pilot experiments and it is reported in all figure legends.
Data exclusions	There was no excluded data.
Replication	Experiments were replicated as noted in the figure legends. All replicates were considered for the analysis.
Randomization	Randomization does not apply for this study.
Blinding	For microscopy experiments, regions of interest were selected on a non-relevant fluorescence channel or in phase contrast, depending on the experiment, to ensure unbiased region selection. For timelapse experiments, positions were randomly selected prior to the beginning of the acquisition. When possible, all the analyses were performed automatically using the same settings for all experimental conditions, to avoid user-derived bias.

Reporting for specific materials, systems and methods

We require information from authors about some types of materials, experimental systems and methods used in many studies. Here, indicate whether each material, system or method listed is relevant to your study. If you are not sure if a list item applies to your research, read the appropriate section before selecting a response.

Materials & experimental systems

n/a	Involved in the study	
☐	☒	Antibodies
☐	☒	Eukaryotic cell lines
☒	☐	Palaeontology and archaeology
☒	☐	Animals and other organisms
☒	☐	Clinical data
☒	☐	Dual use research of concern

Methods

n/a	Involved in the study	
☒	☐	ChIP-seq
☒	☐	Flow cytometry
☒	☐	MRI-based neuroimaging

Antibodies

Antibodies used	All used antibodies and experimental methods are extensively detailed in Materials and Methods
Validation	All antibodies were validated by the commercial vendor for the indicated application (immunofluorescence and western blot)

Eukaryotic cell lines

Policy information about cell lines and Sex and Gender in Research

Cell line source(s)	All cell lines were purchased from ATCC. Primary CAFs were generated in house as described in Glentis at al, Nature Commun 2017.
Authentication	all cell line were authenticated.
Mycoplasma contamination	All cells used were tested for Mycoplasma contamination every 2 weeks. The cell lines used in this study were never found to be contaminated with Mysocplasma.
Commonly misidentified lines (See ICLAC register)	NA
